# Hypothyroidism and meniere's disease: Insight from mendelian randomization study in a European population

**DOI:** 10.1016/j.bjorl.2026.101935

**Published:** 2026-10-31

**Authors:** Yuanzhi Zhu, Jian Luo

**Affiliations:** The First People’s Hospital of Yibin, Department of Otorhinolaryngology, Yibin, Sichuan, China

**Keywords:** Hypothyroidism, Meniere's disease, Causal relationship, Mendelian randomization study

## Abstract

**Objectives:**

Several observational studies have identified a link between hypothyroidism and Meniere's Disease (MD), but there is currently no reliable evidence of a causal relationship between the two conditions. We employed a novel data analysis approach, known as Mendelian randomization, to evaluate the potential causal link between hypothyroidism and MD.

**Methods:**

Summary statistics for hypothyroidism and MD were obtained from the FinnGen Consortium, which included a total of 606,623 participants. The Inverse Variance Weighting (IVW) method served as the main approach for evaluating the causal link between hypothyroidism and MD, supplemented by four other methods. To confirm the reliability and robustness of the results, we applied Cochran's*Q*-test, the MR-Egger intercept test, and the leave-one-out analysis. Bidirectional MR analysis was also performed to address potential reverse causality bias.

**Results:**

The random-effects IVW method showed no causal relationship between hypothyroidism and MD (OR = 0.972, 95% CI 0.913–1.034, p = 0.367). In a reverse analysis, there was no evidence that MD had an effect on hypothyroidism (OR = 1.021, 95% CI: 0.998–1.046, p = 0.076). The findings from the four additional Mendelian randomization methods aligned with the results obtained using the IVW approach. Furthermore, sensitivity analyses showed no horizontal pleiotropy or heterogeneity.

**Conclusion:**

This MR study demonstrated no genetic causal relationship between hypothyroidism and MD, and hypothyroidism is not a risk factor for MD.

**Level of evidence:**

Level 2.

## Highlights

• Mendelian randomization explored hypothyroidism-MD link.
• No evidence of causal association was identified.
• Genetic liability to hypothyroidism does not affect MD.

## Introduction

Meniere's disease is an inner ear disease characterized by recurrent episodes of rotational vertigo, fluctuating sensorineural hearing loss, tinnitus, and aural fullness.^1^^,^^2^ The disease typically begins in one ear, but may spread to both ears as it progresses. Most patients are between 40 and 60 years old, and women are more susceptible than men. According to a Finnish study, 513 people per 100,000 were affected by Meniere's disease.^3^ Although endolymphatic hydrops is the most common histopathological finding in patients with MD, its exact mechanism remains unclear.^4^ MD is a multifactorial disease. Current studies believe that the occurrence of the disease is related to multiple factors such as imbalance in endolymph production and absorption, immune damage, endolymphatic sac dysfunction, viral infection, and genetic susceptibility.^5^ Although the mechanism of MD is still ambiguous, several studies have shown that there may be an association between hypothyroidism and MD.^6^, ^7^, ^8^

An observational study showed that among 35 patients clinically diagnosed with Meniere's disease, 12 were diagnosed with hypothyroidism; all MD patients with hypothyroidism received 12-weeks of oral thyroid supplementation, and the symptoms of MD were subjectively improved.^6^ A study from South Korea reported a higher prevalence of hypothyroidism in the MD group compared to the control group, and this study revealed that hypothyroidism was linked to an increased risk of MD, with an adjusted Odds Ratio (OR) of 1.21 (95% CI 1.02–1.44).^7^ A study by Lin et al. showed that the overall incidence of MD was higher in individuals with hypothyroidism compared to those in the control group; after thyroid hormone treatment, the risk of Meniere's disease was reduced and did not differ from that in patients without hypothyroidism.^8^ The above studies suggested that there may be a causal relationship between hypothyroidism and Meniere's disease. However, since observational studies are easily affected by reverse causality or confounding variables,^9^ it is impossible to make a causal inference between hypothyroidism and Meniere's disease. Although randomized controlled trials can be used for causal inference, the low prevalence of MD makes it difficult to conduct large-scale RCTs. Therefore, it is urgent to find new statistical methods to explore the causal association between hypothyroidism and Meniere's disease.

Mendelian Randomization (MR) is an epidemiological tool that constructs models and infers and evaluates causal effects by screening Single-Nucleotide Polymorphisms (SNPs) that are strongly associated with exposure as Instrumental Variables (IV).^10^ During the slow division process, since the alleles of SNPs are randomly assigned to sperm/eggs, MR studies are effective in minimizing the influence of reverse causality and confounding factors on the outcomes.^11^ In recent years, Genome-Wide Association Studies (GWAS) have made significant progress and accumulated up to millions of associations between genetic variants and phenotypes. MR studies that use published GWAS data for causal inference can save a lot of research costs. Therefore, MR studies were widely used by researchers.^12^, ^13^, ^14^

This study aimed to investigate the potential genetic causal relationship between hypothyroidism and MD by performing a two-sample bidirectional MR analysis using the GWAS statistical summary of hypothyroidism and MD.

## Methods

### Study design

We conducted a two-sample bidirectional MR analysis to explore the bidirectional causal relationship between hypothyroidism and MD. This study was based on the following three theoretical assumptions: 1) The selected IVs should be strongly correlated with the exposure^15^; (2) The IVs should be independent of the confounding factors associated with the selected exposure and outcome;^15^ (3) The selected IVs influence the outcome solely through the exposure.^15^ Fig. 1 briefly described the process of this MR study.

**Fig. 1:**
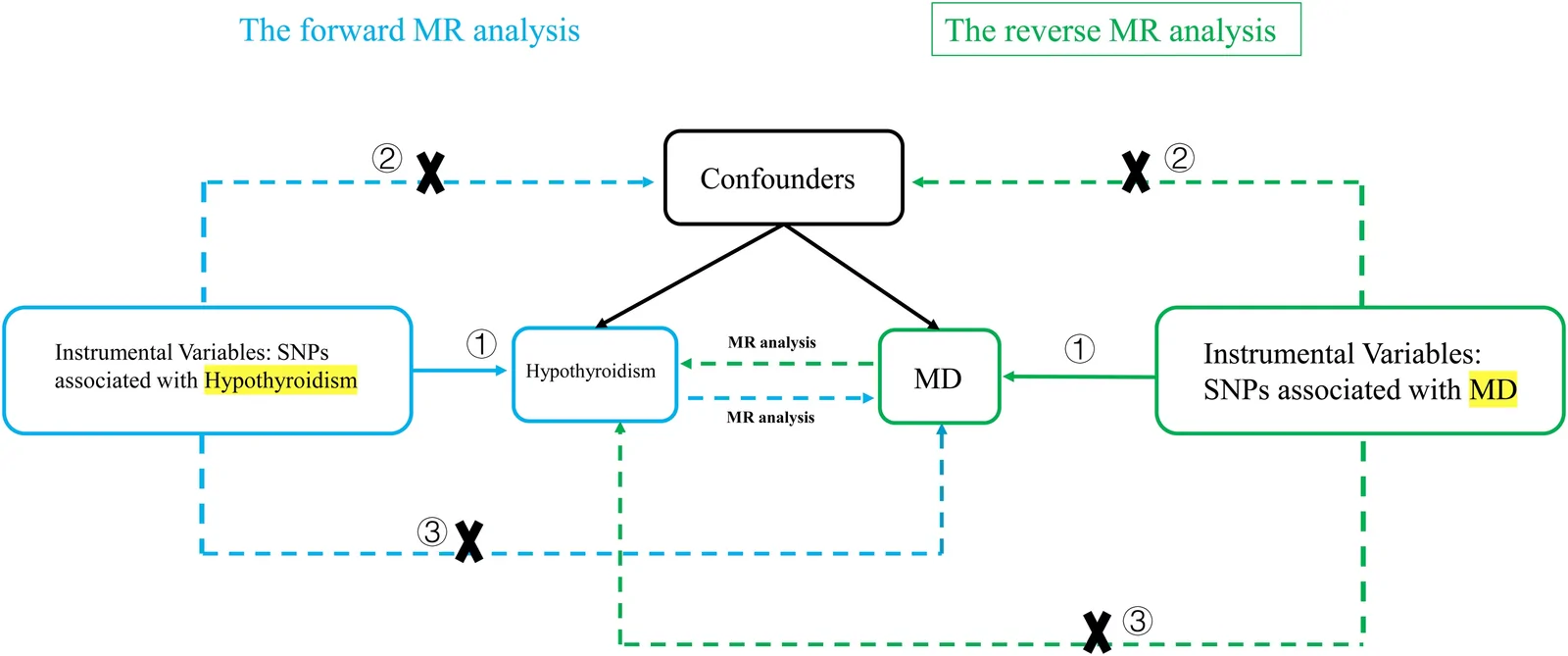
Description of this bidirectional MR study design ①: Genetic variants are associated with exposure; ②: Genetic variants are independent of confounders; ③: Genetic variants affect outcome only through risk factors.

### Data resources

We obtained the GWAS dataset for hypothyroidism from the 12th version of the Finngen Consortium, including 128,104 participants (16,150 cases, 111,954 controls).^16^ The GWAS dataset for MD also came from the 12th version of the Finngen Consortium, including 478,519 participants (3680 cases, 474,839 controls).^16^ Since all participants were of European origin, the inference of the causal relationship between hypothyroidism and MD would not be affected by ancestry.

### Instrumental variable selection

In the forward MR analysis, we selected SNPs strongly associated with hypothyroidism as instrumental variables based on strict criteria (correlation: p < 5 × 10^−8^, *F* > 10, independence: *R*^2^ < 0.001, *kb* = 10,000). However, in the reverse MR analysis, only two SNPs strongly associated with MD were identified based on strict criteria (correlation: p < 5 × 10^−8^, *F* > 10, independence: *R*^2^ < 0.001, *kb* = 10,000). Therefore, to include as many instrumental variables as possible, SNPs associated with MD were screened out using p < 5 × 10^−6^ as the criterion in the reverse MR analysis. In addition, all palindromic SNPs and SNPs associated with both confounders and the outcome were excluded.

### Statistical methods

This MR analysis employed five approaches to gauge the causal link between hypothyroidism and MD. Among them, the Inverse Variance Weighted (IVW) method served as the primary tool for evaluating the potential causal effect of hypothyroidism on MD. Because IVW assumes that all SNPs used are true genetic instruments and calculates the inverse variance weighted average of the ratio estimates from two or more instruments, it can produce the most accurate estimates.^17^ Fixed-effect IVW may produce overly precise results in the presence of heterogeneity, so we used random-effect IVW to gauge the causal relationship between hypothyroidism and MD. MR-Egger regression, weighted median, simple model, and weighted model methods were applied as supplementary methods to further assess the causal relationship between hypothyroidism and MD. To ensure the reliability and robustness of the results of the causal relationship assessment between hypothyroidism and MD, we conducted multiple sensitivity analyses. In this study, the Cochran's *Q*-test was conducted to evaluate the heterogeneity among individual SNPs. To detect horizontal pleiotropy, the MR-Egger regression intercept was then applied; a p-value greater than 0.05 suggested no evidence of horizontal pleiotropy.^18^ In addition, we used a leave-one-out approach to test the robustness of our results and to gauge whether any single SNP significantly influenced the causal effect estimate. Inverse MR analysis was performed using the same methods as described above. Results were expressed using Odds Ratios (ORs) and 95% Confidence Intervals (95% CIs), with the difference considered statistically significant when p-value was less than 0.05. We performed the entire MR analysis using the “TwoSampleMR” package in R Studio (version 4.3.0).

## Results

### The casual effect of hypothyroidism on MD

After multiple rounds of screening, 39 SNPs were ultimately identified as instrumental variables for evaluating the causal effect of hypothyroidism on MD. Basic information was provided in Supplementary Table 1. The F-statistics for individual SNPs ranged from 292.87 to 4903.71, indicating that the causal association was minimally influenced by weak instrumental variables. Random-effects IVW analysis revealed no significant evidence supporting a causal effect of hypothyroidism on MD (OR = 0.972, 95% CI: 0.913–1.206, p = 1.034). MR-Egger regression, weighted median, simple model, and weighted model methods also failed to support a causal effect of hypothyroidism on MD. The results of these five methods were shown in Table 1. Cochran's *Q*-test showed no heterogeneity among the SNPs (p = 0.608). The intercept of the MR-Egger regression was not significantly different from 0 (p = 0.885), indicating no horizontal pleiotropy among the SNPs. Furthermore, leave-one-out analysis showed that no single SNP was found to have a potential impact on the MR analysis (Fig. 2).

**Table 1:** The MR results by five methods.

Exposure	Outcome	SNP(n)	Method	β	se	OR	OR 95% CI		p-Value
Hypothyroidism	Meniere's disease	39	MR Egger	−0.019	0.070	0.981	0.854	1.126	0.784
			Weighted median	−0.034	0.047	0.966	0.882	1.059	0.4464
			Inverse variance weighted	−0.029	0.032	0.972	0.913	1.034	0.367
			Simple mode	0.009	0.084	1.257	0.857	1.188	0.916
			Weighted mode	−0.014	0.053	0.986	0.888	1.094	0.794

**Fig. 2:**
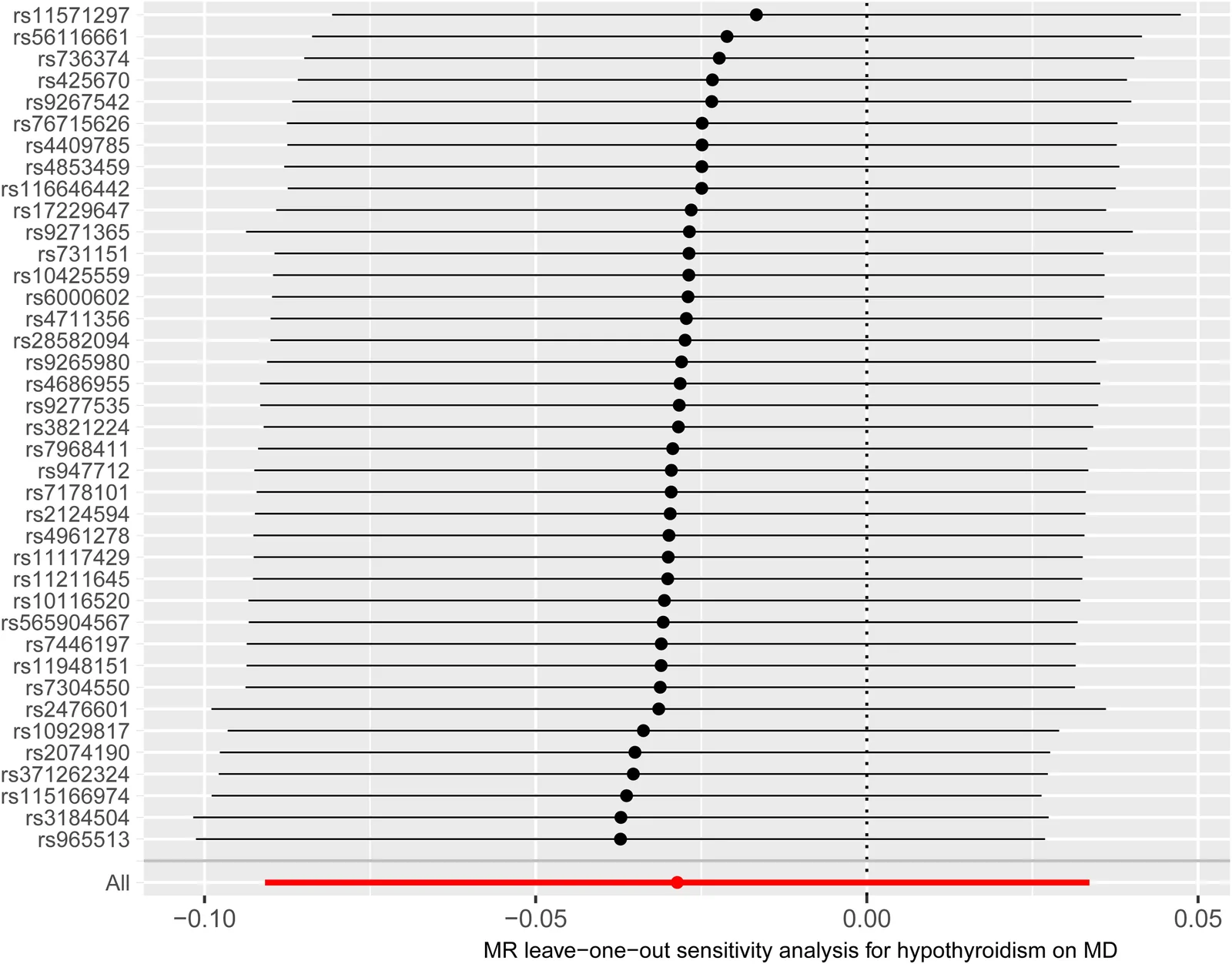
MR analysis.

### The casual effect of MD on hypothyroidism

Based on the instrumental variable selection criteria, 20 SNPs were ultimately selected as instrumental variables to gauge the causal effect of MD on hypothyroidism. Basic information was provided in Supplementary Table 2. The F statistics for individual SNPs ranged from 47.18 to 161.44, indicating that the causal association was minimally influenced by weak instrumental variables. Random-effects IVW analysis revealed no significant evidence supporting a causal effect of MD on hypothyroidism (OR = 1.021, 95% CI: 0.998–1.206, p = 1.046). Furthermore, the results of MR-Egger regression, weighted median, simple model, and weighted model methods were consistent with those obtained using the IVW method. The results of these five methods were shown in Table 2. The Cochran's *Q*-test showed no heterogeneity among the SNPs (p = 0.712). The intercept of the MR-Egger regression was not statistically significantly different from 0 (p = 0.939), indicating no horizontal pleiotropy among the SNPs. Furthermore, the results of the leave-one-out analysis confirmed the robustness of the results of this analysis (Fig. 3).

**Table 2:** The MR results by five methods.

Exposure	Outcome	SNP(n)	Method	β	se	OR	OR 95% CI		p-Value
Meniere's disease	Hypothyroidism	20	MR Egger	0.022	0.015	1.022	0.992	1.053	0.166
			Weighted median	0.023	0.015	1.023	0.993	1.054	0.134
			Inverse variance weighted	0.021	0.012	1.021	0.998	1.046	0.076
			Simple mode	0.008	0.046	1.008	0.922	1.103	0.863
			Weighted mode	0.022	0.014	1.022	0.995	1.050	0.126

**Fig. 3:**
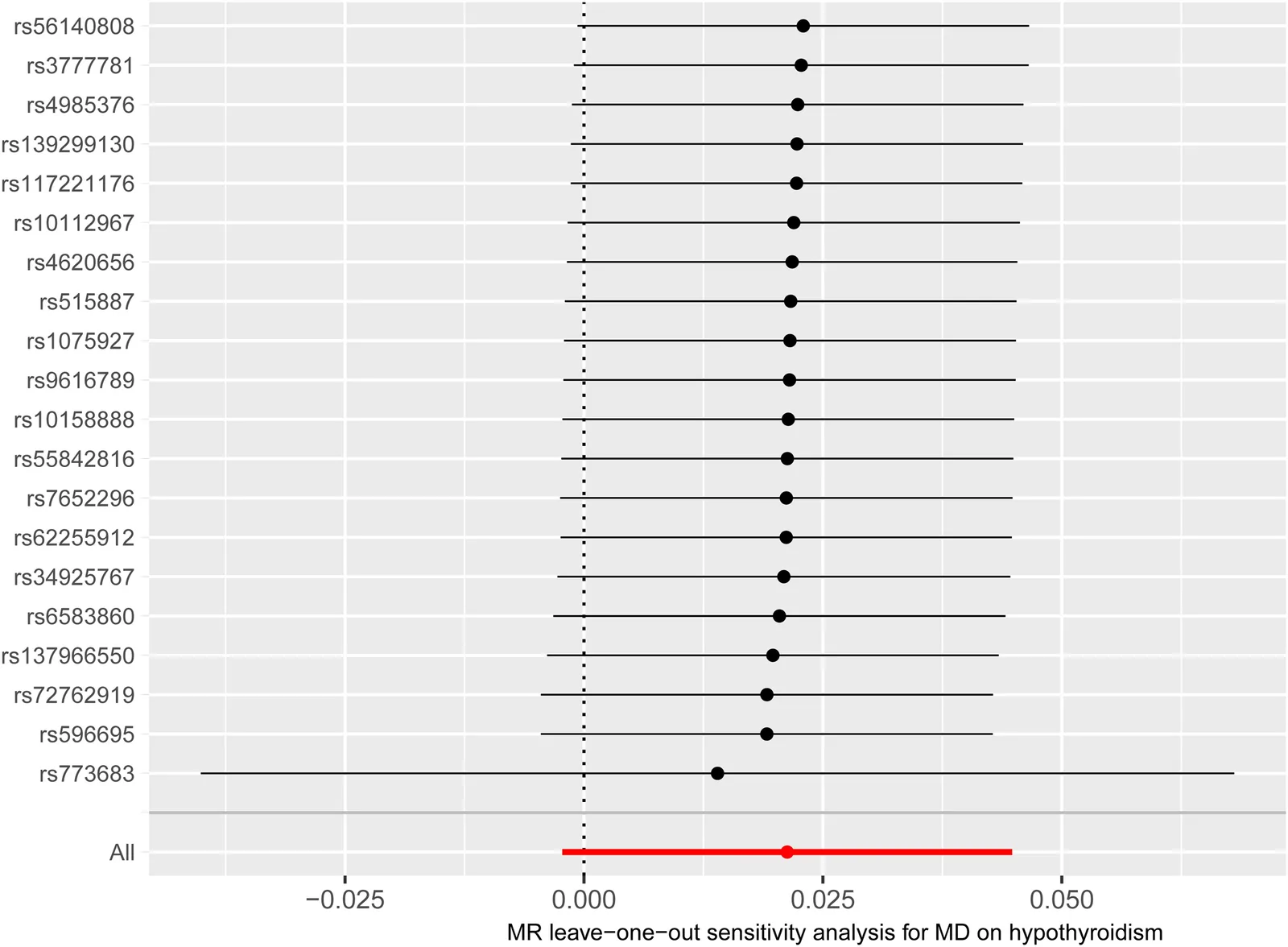
Analysis.

## Discussion

MD is a chronic disease that not only causes varying degrees of functional impairment and increases the emotional burden on patients, but also places an economic burden on families and society.^1^ In order to reduce the impact of MD on patients, more attention should be paid to the causes of MD. Although some studies have shown a correlation between hypothyroidism and MD,^6^^,^^8^^,^^19^ due to the limitations of observational studies, it is currently impossible to determine whether there is a causal relationship between hypothyroidism and MD. To the best of our knowledge, this is the first study to apply MR analysis to investigate the potential causal relationship between hypothyroidism and MD. The findings indicated no causal association, suggesting that hypothyroidism does not influence the development of MD. Negative results are not meaningless, and the results of many observational studies have been overturned by many MR studies.^20^, ^21^, ^22^

An observational study showed that among patients with hypothyroidism, 43% had hearing impairment, 7% had tinnitus, and 29.1% had vertigo; the incidence of these symptoms was linearly correlated with the severity of hypothyroidism.^23^ Bakdounes et al.^24^ showed that the incidence of MD in patients with hypothyroidism was 14.3%, which was higher than the incidence in the general population in Finland.^3^ Another observational study involving 27,050 subjects showed that the incidence of MD in the hypothyroid group was higher than that in the non-hypothyroid group, and Cox model analysis showed that hypothyroidism was an independent risk factor for MD.^8^ Previous studies have suggested that hypothyroidism may cause patients to develop MD symptoms by affecting metabolic processes.^25^, ^26^, ^27^ However, there is currently no conclusive evidence that hypothyroidism causes the occurrence of MD. In this study, we conducted MR analysis on a large sample population to explore this issue and ultimately found no evidence of a causal link between hypothyroidism and MD; hypothyroidism was not a risk factor for MD.

Our study has several strengths worth mentioning. First, we conducted the largest genome-wide association study of MD to date using a two-sample Mendelian randomization analysis, which improved the accuracy of assessing the causal relationship between hypothyroidism and MD while saving considerable time and financial costs. Second, compared with traditional observational studies, Mendelian randomization studies overcome the influence of reverse causation and confounding factors on the study results. Finally, we conducted multiple sensitivity analyses to verify the reliability and robustness of the MR analysis results.

However, our study has several limitations. First, because the GWAS data for hypothyroidism and MD were collected from European populations, our findings cannot be generalized to other populations. Second, due to the lack of individual data, we were unable to conduct stratified analyses by MD severity. Third, due to insufficient detailed information on participant overlap between the two published GWAS pooled datasets, it is difficult to assess potential bias due to participant overlap.

## Conclusion

In conclusion, our MR analysis found no evidence of a genetic causal relationship between hypothyroidism and Meniere’s Disease (MD), suggesting that hypothyroidism is unlikely to be a primary causal factor in the development of MD. These findings do not support a genetically mediated causal effect of hypothyroidism on MD, while appropriate thyroid management remains important for the overall clinical care of patients with MD.

## Funding

No.

## Data availability

The authors declare that all data are available in repository.

## Declaration of competing interest

The authors declare no conflicts of interest.

## References

[1] Hoskin J.L. (2022). Ménière’s disease: new guidelines, subtypes, imaging, and more. Curr Opin Neurol.

[2] Basura G.J., Adams M.E., Monfared A. (2020). Clinical practice guideline: Ménière’s disease. Otolaryngol Head Neck Sur.

[3] Havia M., Kentala E., Pyykkö I. (2005). Prevalence of Menière’s disease in general population of Southern Finland. Otolaryngol Head Neck Surg.

[4] Rizk H.G., Mehta N.K., Qureshi U. (2022). Pathogenesis and etiology of Ménière disease: a scoping review of a century of evidence. JAMA Otolaryngol Head Neck Surg.

[5] Mohseni-Dargah M., Falahati Z., Pastras C. (2023). Meniere’s disease: pathogenesis, treatments, and emerging approaches for an idiopathic bioenvironmental disorder. Environ Res.

[6] Santosh U.P., Rao M.S. (2016). Incidence of hypothyroidism in Meniere’s disease. J Clin Diagn Res.

[7] Kim S.Y., Song Y.S., Wee J.H., Min C., Yoo D.M., Choi H.G. (2020). Association between Ménière’s disease and thyroid diseases: a nested case-control study. Sci Rep.

[8] Lin W.L., Chen C.Y., Hsu T.Y., Chen W.K., Lin C.L., Chen H.C. (2019). Hypothyroidism is an independent risk factor for Menière’s disease: a population-based cohort study. Medicine (Baltimore).

[9] Boyko E.J. (2013). Observational research—opportunities and limitations. J Diabetes Complications.

[10] Emdin C.A., Khera A.V., Kathiresan S. (2017). Mendelian randomization. JAMA.

[11] Bai R., Ren L., Guo J. (2023). The causal relationship between pure hypercholesterolemia and psoriasis: a bidirectional, two-sample Mendelian randomization study. Skin Res Technol.

[12] Gnatiuc Friedrichs L., Kuri-Morales P., Trichia E. (2025). A Mendelian randomization study of the effect of body mass index on 52 causes of death among 125 000 Mexican adults with admixed ancestry. Int J Epidemiol.

[13] Sun J., Fan G., Hu L., Qu Z.H., Jiang H. (2025). Epigenetic age acceleration and allergic diseases: a bidirectional two-sample Mendelian randomization study. Clin Epigenetics.

[14] Jia M.J., Yin H.F., Liang Y.C., Jiang F., Li H.L. (2024). Effect of trace elements and nutrients on 21 autoimmune diseases: a Mendelian randomization study. Front Immunol.

[15] Davies N.M., Holmes M.V., Davey Smith G. (2018). Reading Mendelian randomisation studies: a guide, glossary, and checklist for clinicians. BMJ.

[16] FinnGen, Kurki M.I., Karjalainen J. (2023). FinnGen provides genetic insights from a well-phenotyped isolated population. Nature.

[17] Zhu Z., Zhang F., Hu H. (2016). Integration of summary data from GWAS and eQTL studies predicts complex trait gene targets. Nat Genet.

[18] Olze H., Zuberbier T. (2011). Comorbidities between nose and skin allergy. Curr Opin Allergy Clin Immunol.

[19] Lai R., Tang L., Zhu T., Li Q. (2025). Association between Ménière’s disease and thyroid disease: a systematic review and meta-analysis. Clin Otolaryngol.

[20] Gao W., Ma P., Wang Z. (2024). Restriction of salt, alcohol and coffee intake and Ménière’s disease: insight from Mendelian randomization study. Front Nutr.

[21] Wu H., Wu L., Zhang Q., Li C., Li H.Y., Zhang B.F. (2024). No causal relationship between serum vitamin D levels and alcoholiC. liver disease: a two-sample bidirectional Mendelian randomization study. Front Nutr.

[22] Xie J., Yang M., Yu H. (2022). No evidence to support a causal relationship between circulating adiponectin levels and ankylosing spondylitis: a bidirectional two-sample Mendelian randomization study. Genes.

[23] Bhatia P.L., Gupta O.P., Agrawal M.K., Mishr S.K. (1977). Audiological and vestibular function tests in hypothyroidism. Laryngoscope.

[24] Bakdounes A., Akashe N., Alchallah M.O. (2022). Prevalence of Ménière’s Disease in Syrian Patients with hypothyroidism: cross-sectional study. Ann Med Surg (Lond).

[25] Shambaugh G.E. (1959). Endocrine aspects of Meniere’s disease. Laryngoscope.

[26] Powers W.H. (1972). Metabolic aspects of Meniere’s disease. Laryngoscope.

[27] Pulec J.L. (1972). Meniere’s disease: results of a two and one-half-year study of etiology, natural history and results of treatment. Laryngoscope.

