# Supplementary material for: Hypothyroidism and meniere's disease: Insight from mendelian randomization study in a European population
Source: Braz J Otorhinolaryngol. 2026 Oct 31;93(1):101935. doi: 10.1016/j.bjorl.2026.101935 (PMC13743938; doi:10.1016/j.bjorl.2026.101935)
Supplement: Supplementary file 1 [file mmc1.docx]

BJORL-D-26-00135_Supplementary Material

**Supplementary Table 1** Detailed information of selected SNPs for MR analysis of the causal effect of hypothyroidism on Meniere's disease.

| **SNP** | **Effect allele** | **Other allele** | **Hypothyroidism** | | | | **Meniere's disease** | | | |
| --- | --- | --- | --- | --- | --- | --- | --- | --- | --- | --- |
|  |  |  | **EAF** | **β** | **SE** | **p-value** | **EAF** | **β** | **SE** | **p-value** |
| rs10116520 | G | A | 0.402609 | 0.101477 | 0.0145231 | 2.80E-12 | 0.402075 | 0.007857 | 0.024017 | 0.743546 |
| rs10425559 | G | A | 0.597869 | -0.0983 | 0.0145256 | 1.31E-11 | 0.598582 | 0.012806 | 0.024039 | 0.594224 |
| rs10929817 | C | T | 0.383911 | -0.09893 | 0.0146659 | 1.53E-11 | 0.384874 | -0.02658 | 0.02416 | 0.271253 |
| rs11117429 | G | C | 0.41399 | -0.07929 | 0.0144934 | 4.48E-08 | 0.413949 | -0.00733 | 0.023888 | 0.759076 |
| rs11211645 | G | A | 0.416059 | 0.081584 | 0.0144729 | 1.73E-08 | 0.414648 | 0.00763 | 0.023965 | 0.750186 |
| rs115166974 | T | C | 0.026325 | 0.267576 | 0.0427222 | 3.77E-10 | 0.0264 | 0.146849 | 0.074279 | 0.048041 |
| rs11571297 | C | T | 0.370299 | -0.1819 | 0.014871 | 2.10E-34 | 0.371784 | 0.041567 | 0.024322 | 0.08744 |
| rs116646442 | G | C | 0.00283 | 0.679915 | 0.121217 | 2.03E-08 | 0.002594 | -0.31029 | 0.233349 | 0.183612 |
| rs11948151 | T | C | 0.319985 | 0.083633 | 0.0152313 | 4.00E-08 | 0.319931 | 0.015726 | 0.025178 | 0.532248 |
| rs17229647 | C | T | 0.054664 | 0.188107 | 0.0305819 | 7.70E-10 | 0.055198 | -0.03506 | 0.052034 | 0.500392 |
| rs2074190 | G | A | 0.292874 | 0.096505 | 0.0155441 | 5.35E-10 | 0.292446 | 0.040609 | 0.025884 | 0.116672 |
| rs2124594 | C | T | 0.315579 | -0.09585 | 0.0153832 | 4.64E-10 | 0.317508 | -0.00421 | 0.025315 | 0.867784 |
| rs2476601 | G | A | 0.85001 | -0.40568 | 0.0186759 | 1.27E-104 | 0.852386 | 0.005209 | 0.033199 | 0.875308 |
| rs28582094 | G | A | 0.26216 | 0.089481 | 0.0160294 | 2.37E-08 | 0.262595 | -0.01181 | 0.026691 | 0.658264 |
| rs3184504 | C | T | 0.593933 | -0.20115 | 0.0144165 | 3.04E-44 | 0.593064 | -0.01662 | 0.023962 | 0.487898 |
| rs371262324 | C | T | 0.032706 | 0.224845 | 0.0394614 | 1.21E-08 | 0.033059 | 0.12608 | 0.067645 | 0.062343 |
| rs3821224 | G | C | 0.296923 | -0.09027 | 0.0157462 | 9.87E-09 | 0.297291 | 0.003866 | 0.025817 | 0.88098 |
| rs425670 | T | C | 0.604288 | 0.080678 | 0.0145887 | 3.20E-08 | 0.60401 | -0.03967 | 0.024057 | 0.099129 |
| rs4409785 | C | T | 0.166644 | 0.108425 | 0.0188616 | 9.01E-09 | 0.166419 | -0.03686 | 0.031601 | 0.243447 |
| rs4686955 | G | A | 0.381924 | -0.14749 | 0.0147975 | 2.12E-23 | 0.383302 | 0.005872 | 0.02419 | 0.8082 |
| rs4711356 | G | A | 0.009921 | 0.490905 | 0.0664767 | 1.53E-13 | 0.009876 | -0.05386 | 0.12093 | 0.656044 |
| rs4853459 | C | T | 0.767306 | -0.13965 | 0.016585 | 3.76E-17 | 0.768639 | 0.023955 | 0.027877 | 0.390167 |
| rs4961278 | A | G | 0.620522 | -0.096 | 0.0146787 | 6.16E-11 | 0.621498 | -0.00475 | 0.024278 | 0.844725 |
| rs56116661 | T | C | 0.22114 | -0.10216 | 0.0173018 | 3.53E-09 | 0.223084 | 0.060476 | 0.028279 | 0.032476 |
| rs565904567 | T | C | 0.075782 | 0.144717 | 0.0264198 | 4.31E-08 | 0.075673 | 0.024459 | 0.044554 | 0.583027 |
| rs6000602 | A | C | 0.187741 | 0.127597 | 0.017992 | 1.32E-12 | 0.186694 | -0.01494 | 0.030151 | 0.620236 |
| rs7178101 | C | T | 0.654208 | -0.08273 | 0.0148944 | 2.79E-08 | 0.656741 | -0.0043 | 0.024761 | 0.86207 |
| rs7304550 | A | G | 0.280804 | -0.09091 | 0.0160523 | 1.49E-08 | 0.280823 | -0.0167 | 0.026374 | 0.526492 |
| rs731151 | A | G | 0.203547 | 0.095932 | 0.0174974 | 4.19E-08 | 0.20332 | -0.0183 | 0.029284 | 0.531987 |
| rs736374 | A | G | 0.349201 | 0.090451 | 0.0148143 | 1.02E-09 | 0.349673 | -0.04426 | 0.024646 | 0.072505 |
| rs7446197 | G | A | 0.672019 | 0.084016 | 0.015328 | 4.22E-08 | 0.670599 | 0.015366 | 0.025119 | 0.540711 |
| rs76715626 | C | T | 0.060244 | 0.192928 | 0.0290794 | 3.26E-11 | 0.059486 | -0.05346 | 0.050151 | 0.286461 |
| rs7968411 | A | G | 0.469009 | 0.079938 | 0.0142911 | 2.22E-08 | 0.468219 | 0.002365 | 0.023555 | 0.920019 |
| rs9265980 | A | G | 0.09358 | 0.133704 | 0.0240279 | 2.63E-08 | 0.093342 | -0.01158 | 0.040475 | 0.774838 |
| rs9267542 | T | C | 0.37831 | -0.14236 | 0.0148551 | 9.41E-22 | 0.376949 | 0.024954 | 0.024411 | 0.306662 |
| rs9271365 | G | T | 0.361344 | 0.283174 | 0.0145511 | 2.37E-84 | 0.364213 | -0.01141 | 0.024419 | 0.640211 |
| rs9277535 | G | A | 0.195752 | -0.16542 | 0.0182982 | 1.56E-19 | 0.197828 | 0.006235 | 0.029463 | 0.832406 |
| rs947712 | T | C | 0.382508 | -0.11598 | 0.0147556 | 3.85E-15 | 0.384139 | -0.00108 | 0.024283 | 0.964526 |
| rs965513 | G | A | 0.652673 | 0.185853 | 0.0151449 | 1.29E-34 | 0.6519 | 0.020978 | 0.02469 | 0.395518 |

**Supplementary Table 2** Detailed information of selected SNPs for MR analysis of the causal effect of Meniere's disease on hypothyroidism.

| **SNP** | **Effect allele** | **Other allele** | **Meniere's disease** | | | | | **Hypothyroidism** | | | |
| --- | --- | --- | --- | --- | --- | --- | --- | --- | --- | --- | --- |
|  |  |  | **EAF** | **β** | | **SE** | **p-value** | **EAF** | **β** | **SE** | **p-value** |
| rs10112967 | C | T | 0.531067 | 0.124699 | | 0.023668 | 1.37E-07 | 0.529917 | -0.00524 | 0.014424 | 0.716581 |
| rs10158888 | A | G | 0.566718 | 0.112857 | | 0.023816 | 2.15E-06 | 0.565892 | 0.000956 | 0.01434 | 0.946865 |
| rs1075927 | G | A | 0.15505 | -0.1639 | | 0.034041 | 1.47E-06 | 0.155672 | 0.001351 | 0.019889 | 0.945825 |
| rs117221176 | T | G | 0.059782 | 0.224842 | | 0.046515 | 1.34E-06 | 0.059499 | -0.02288 | 0.03032 | 0.450504 |
| rs137966550 | A | G | 0.305427 | -0.11953 | | 0.02583 | 3.70E-06 | 0.304074 | -0.02313 | 0.015459 | 0.134677 |
| rs139299130 | T | C | 0.056992 | -0.27142 | | 0.055081 | 8.32E-07 | 0.056641 | 0.019014 | 0.030934 | 0.538769 |
| rs34925767 | G | A | 0.055774 | 0.299859 | | 0.046434 | 1.06E-10 | 0.056779 | 0.013872 | 0.030667 | 0.651027 |
| rs3777781 | A | T | 0.302932 | -0.19188 | | 0.026157 | 2.20E-13 | 0.303775 | 0.008392 | 0.015488 | 0.587933 |
| rs4620656 | A | T | 0.999603 | -1.66969 | | 0.352445 | 2.16E-06 | 0.999596 | 0.262909 | 0.372141 | 0.479891 |
| rs4985376 | A | G | 0.618263 | -0.11016 | | 0.023918 | 4.11E-06 | 0.616971 | 0.012488 | 0.014693 | 0.395359 |
| rs515887 | C | A | 0.772494 | -0.12903 | | 0.027425 | 2.54E-06 | 0.772502 | 0.002948 | 0.017067 | 0.862849 |
| rs55842816 | T | C | 0.047325 | -0.28486 | | 0.060518 | 2.51E-06 | 0.047969 | -0.00565 | 0.034078 | 0.868232 |
| rs56140808 | C | T | 0.270826 | 0.11938 | | 0.025975 | 4.31E-06 | 0.271369 | -0.02258 | 0.016083 | 0.160315 |
| rs596695 | A | G | 0.367923 | -0.11957 | | 0.024669 | 1.26E-06 | 0.367784 | -0.02947 | 0.014825 | 0.046831 |
| rs62255912 | C | T | 0.012166 | 0.432526 | | 0.093561 | 3.78E-06 | 0.012091 | 0.016157 | 0.066028 | 0.806689 |
| rs6583860 | A | G | 0.324111 | 0.130939 | | 0.024694 | 1.14E-07 | 0.32403 | 0.012752 | 0.015283 | 0.404068 |
| rs72762919 | T | C | 0.101306 | -0.20583 | | 0.041123 | 5.58E-07 | 0.101798 | -0.04457 | 0.023846 | 0.061593 |
| rs7652296 | G | A | 0.496844 | 0.12744 | | 0.023399 | 5.14E-08 | 0.498006 | 0.003671 | 0.014253 | 0.796767 |
| rs773683 | A | T | 0.002058 | 1.28773 | | 0.269404 | 1.75E-06 | 0.222449 | 0.029577 | 0.01717 | 0.084953 |
| rs9616789 | A | G | 0.040063 | | -0.3185 | 0.066218 | 1.51E-06 | 0.039542 | 0.000339 | 0.03688 | 0.992672 |
